# Consumption of a High-Fat Diet Alters Perineuronal Nets in the Prefrontal Cortex

**DOI:** 10.1155/2018/2108373

**Published:** 2018-04-23

**Authors:** P. M. Dingess, J. H. Harkness, M. Slaker, Z. Zhang, S. S. Wulff, B. A. Sorg, T. E. Brown

**Affiliations:** ^1^Neuroscience Program, University of Wyoming, Laramie, WY 82071, USA; ^2^Department of Integrative Physiology and Neuroscience, Washington State University, Vancouver, WA 98686, USA; ^3^Department of Pharmacology and Toxicology, Medical College of Wisconsin, Milwaukee, WI 53226, USA; ^4^Department of Zoology and Physiology, University of Wyoming, Laramie, WY 82071, USA; ^5^Department of Statistics, University of Wyoming, Laramie, WY 82071, USA; ^6^School of Pharmacy, University of Wyoming, Laramie, WY 82071, USA

## Abstract

A key factor in the development of obesity is the overconsumption of fatty foods, which, in addition to facilitating weight gain, alters neuronal structures within brain reward circuitry. Our previous work demonstrates that sustained consumption of a high-fat diet (HFD) attenuates spine density in the prefrontal cortex (PFC). Whether HFD promotes structural adaptation among inhibitory cells of the PFC is presently unknown. One structure of interest is the perineuronal net (PNN), a specialized extracellular matrix surrounding, primarily, parvalbumin-containing GABAergic interneurons. PNNs contribute to synaptic stabilization, protect against oxidative stress, regulate the ionic microenvironment within cells, and modulate regional excitatory output. To examine diet-induced changes in PNNs, we maintained rats on one of three dietary conditions for 21 days: ad libitum chow, ad libitum 60% high fat (HF-AL), or limited-access calorically matched high fat (HF-CM), which produced no significant change in weight gain or adiposity with respect to chow controls. The PNN “number” and intensity were then quantified in the prelimbic (PL-PFC), infralimbic (IL-PFC), and ventral orbitofrontal cortex (OFC) using *Wisteria floribunda* agglutinin (WFA). Our results demonstrated that fat exposure, independent of weight gain, induced a robust decrease in the PNN intensity in the PL-PFC and OFC and a decrease in the PNN number in the OFC.

## 1. Introduction

While there are many behavioral, environmental, and even genetic circumstances that contribute to the development of obesity [1], the overconsumption of fatty foods is a major catalyst [2, 3]. However, knowledge that high-fat (HF) foods facilitate weight gain and increase the risk of disease has not deterred patients from engaging in maladaptive feeding behaviors [4]. Studies demonstrate that demographic factors such as age and gender influence poor adherence to diet and exercise regimens, but that poor adherence is especially prevalent among individuals who are *already* overweight or obese [5, 6]. This raises an interesting question—does the consumption of HF food induce changes in reward circuit neurophysiology that impair an individual's ability to make favorable decisions regarding their health?

This question motivated our examination of the prefrontal cortex (PFC), a reward circuit region known to regulate reward-driven decision-making. While several studies indicate that fat exposure elicits structural and functional neuroadaptations in the nucleus accumbens [7–9], a downstream target of the PFC, the exact cellular modifications occurring in the PFC are relatively less understood. Our previous work, demonstrating that rats fed with a HF diet exhibit an attenuation of spine density on pyramidal neurons of the infralimbic prefrontal cortex (IL-PFC) [10], suggests that the PFC may be a critical target of diet-induced structural change.

The PFC is a region of cellular heterogeneity. In addition to pyramidal neurons, the PFC houses parvalbumin-containing GABAergic interneurons, which heavily modulate pyramidal cell excitability [11]. Approximately 75% of these interneurons are covered with a specialized extracellular matrix structure known as the perineuronal net (PNN) [12], which contributes to synaptic stabilization [13], protects against oxidative stress [14], and regulates the ionic microenvironment of cells [15]. When PNNs are enzymatically degraded with chondroitinase ABC in the PFC, GABAergic cell firing decreases [16] and pyramidal cell firing increases [17], providing evidence that PNNs play a key role in maintaining local inhibition. A recent study demonstrates that consumption of a HF diet reduces GABA concentrations in the frontal cortex [18]. Thus, we became interested in the effect of a HF diet on PNNs in the PFC.

To examine the extent to which a HF diet induces changes in PNNs, we maintained rats on one of three dietary conditions for 21 days: ad libitum chow, ad libitum 60% high fat, or limited-access calorically matched high fat, which induced no significant change in weight gain or adiposity with respect to chow controls. The PNN number and intensity were then quantified in the prelimbic PFC (PL-PFC), IL-PFC, and ventral orbitofrontal cortex (OFC). Our results demonstrated that fat exposure, independent of weight gain, induced a significant reduction in the cumulative distribution of normalized PNN intensity in all brain regions analyzed with respect to chow controls. In the PL-PFC and OFC, a fat-induced reduction in mean normalized average intensity was also observed, and in the OFC, fat exposure elicited a decrease in the PNN number. These findings provide further insight into the cellular adaptations that occur with exposure to dietary fat and may therefore guide therapeutic efforts in the treatment of obesity.

## 2. Materials and Methods

### 2.1. Animal Ethics

All procedures in this study were performed in accordance with the National Institutes of Health's *Guidelines for the Care and Use of Laboratory Animals* and with the approval from the Institutional Animal Care and Use Committee (IACUC) at the University of Wyoming. Twenty-three adult male Sprague-Dawley rats, postnatal days 60–80 at the start of dietary manipulation, were obtained from our breeding colony and used for this study. Animals were randomly assigned to one of three experimental conditions (*n* = 7-8/group) and then singly housed in clear plastic cages in a temperature-controlled (25°C) vivarium with ad libitum access to water under a standard 12-hour light/dark cycle (0700–1900). Food access was described below. Three cohorts of animals were used, each with 2-3 animals per dietary condition.

### 2.2. Diet Manipulation

Rats were maintained on one of three dietary conditions in their home cage for three weeks: ad libitum standard chow (chow, *n* = 7), ad libitum 60% high fat (HF-AL, *n* = 8), or limited-access calorically matched 60% high fat (HF-CM, *n* = 8). The duration of diet exposure was selected because it has been previously demonstrated to elicit significant weight gain and adiposity in the HF-AL group [10]. The nutritional content of the chow diet by % kilocalorie was 29% protein, 58% carbohydrate, and 13% fat with a total fuel value of 3.36 kcal/g (Rodent Diet 5001, LabDiet, St. Louis, MO). The nutritional content of the lard-based HF diet by % kilocalorie was 20% protein, 20% carbohydrate, and 60% fat with a total fuel value of 5.24 kcal/g (Research Diets, New Brunswick, NJ). Animals in the HF-CM group were also fed with the 60% HF diet but were restricted such that their daily caloric intake, and therefore weight gain, did not significantly differ from that of chow controls at the time of sacrifice. To ensure that the caloric intake of the chow and HF-CM groups were the same, food consumption was measured each day and averaged for each dietary condition. The total amount of food consumed by the chow group was multiplied by the caloric value of the chow diet (3.36 kcal/g) and then divided by the caloric value of the HF diet (5.24 kcal/g) to estimate the amount of food to be given to the HF-CM group. Rats were weighed every other day to monitor the effectiveness of this regimen. At the time of sacrifice, the right epididymal fat pad was dissected and weighed as a proxy for overall fat accumulation. The weight of this fat pad was compared with the length of the right ulna bone, rather than the body weight, in order to control for differences in body stature.

### 2.3. Perineuronal Net Staining and Quantification

Methods for the quantification of PNNs were described previously [17, 19, 20]. Briefly, animals were anesthetized with isoflurane and euthanized via cardiac perfusion (200 mL 1 M phosphate-buffered saline (PBS) followed by 300 mL 4% paraformaldehyde (PFA) in PBS). Whole brains were extracted and placed into 15 mL of 4% PFA for 24 hr and then 15 mL of 20% sucrose in PBS for 24 hr. After removal from 20% sucrose, brains were immediately frozen with dry ice and placed into −80°C for storage until cryo-sectioning. 30 *μ*m coronal sections of the PFC were obtained using a Leica 3050 cryostat at −20°C. Slices were then rinsed in PBS 3× for 5 min each, 50% alcohol for 30 min, and PBS 3× for 5 min each and incubated and gently rocked in 1 : 500 fluorescein-conjugated *Wisteria floribunda* agglutinin (WFA, Vector Laboratories, Burlingame, CA) in PBS overnight (~20 hr) at 4°C. Following WFA incubation, slices were rinsed 3× in PBS for 10 min each and then mounted on glass slides with ProLong Gold Antifade mountant with DAPI (Vector Laboratories) and stored in the dark at 4°C until the time of imaging.

A Zeiss 710 scanning confocal microscope and Zen imaging software were used to acquire images of the PL-PFC, IL-PFC, and ventral OFC, from which 6–8, 4–6, and 5–7 images were acquired per animal per dietary condition. All images were acquired using a 40x oil immersion objective (NA 0.55) with the same zoom (~10x) under identical acquisition settings. All images were acquired as z-stacks, consisting of 25 optical sections each 1 *μ*m in thickness. Representative images are z-stacks reconstructed as max intensity projections using ImageJ software (NIH). For analysis, sequences of the raw images within the z-stack were projected into a sum slice image with no manipulation to brightness or contrast. Background subtraction from each sum slice image was conducted, and then each visible PNN in the image was assigned as a region of interest (ROI), including the cell body and proximal dendrites. The average intensity above background from each ROI (PNN) was calculated. To account for possible heterogeneity across the cohorts, the data were normalized by dividing the data points by the average intensity of the chow controls in the cohort and multiplying by 100, as has been previously performed [17]. The normalized intensities of all animals were represented by the empirical cumulative distribution, empirical frequency distribution, and average intensity for each of the experimental groups. Differences in the cumulative distributions of the normalized PNN intensities between experimental conditions were then analyzed using the nonparametric Kolmogorov-Smirnov test.

### 2.4. Statistical Analyses

All statistical tests were conducted using Prism 6 (GraphPad Software) using one-way and two-way ANOVA with Tukey's multiple comparisons test or the nonparametric Kolmogorov-Smirnov test. All results are summarized as mean ± standard error of the mean (SEM).

## 3. Results

**Figure 1:**
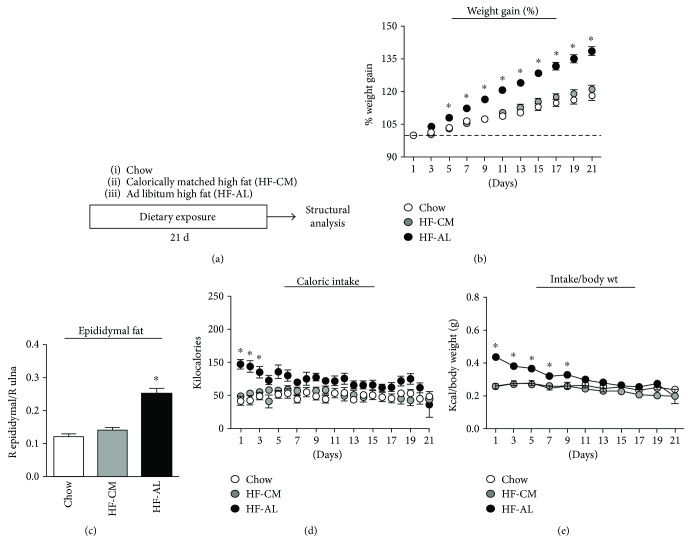
Dietary manipulation. (a) Experimental timeline. Animals were fed with one of three dietary conditions for 21 days, and then tissues were immediately extracted and analyzed. (b) Percent weight gain from baseline in chow (white), HF-CM (gray), and HF-AL (black) groups. (c) Approximate body fat accumulation, expressed as the weight of the right epididymal fat pad over the length of the right ulna bone. (d) Caloric intake across the dietary manipulation. (e) Food consumption expressed as the ratio of kilocalories consumed to body weight. Values represent the mean ± SEM (^∗^*p* < 0.05).

Two-way RM ANOVA with Tukey's multiple comparisons revealed that rats in the HF-AL group gained significantly more weight than rats in the HF-CM and chow groups (% weight gain at 21 d—chow: 118.4 ± 2.15%, HF-CM: 121.1 ± 2.10, and HF-AL: 138.7 ± 2.02; *F*_(2,20)_ = 32.53, *p* < 0.001, Figure 1(b)). There was no difference in mean weight gain between the HF-CM group and chow controls, indicating successful caloric restriction in the HF-CM group. One-way ANOVA revealed a significant increase in the mean epididymal fat pad/ulna ratio in the HF-AL group compared to HF-CM and chow groups (chow: 0.123 ± 0.01, HF-CM: 0.143 ± 0.01, and HF-AL: 0.253 ± 0.01; *F*_(2,12)_ = 82.61, *p* < 0.001, Figure 1(d)). The mean caloric intake did not differ between HF-CM and chow groups. However, caloric intake in the HF-AL group was significantly higher in the first three days of dietary exposure compared to that in the HF-CM and chow groups and remained elevated, though not significantly, throughout the duration of the dietary manipulation (Figure 1(d)). Because consumption remained stable in the HF-AL group but their weight increased, a downward slope was observed when consumption was compared with body weight (Figure 1(e)).

**Figure 2:**
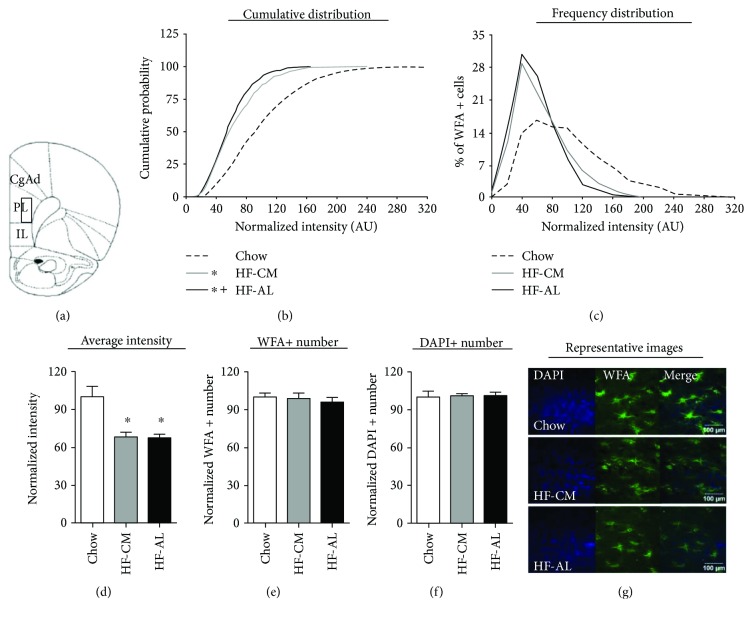
Consumption of a high-fat diet attenuates PNN intensity, but not PNN number, in the prelimbic prefrontal cortex (PL-PFC). (a) Schematic of the region analyzed. (b) Normalized PNN intensity expressed as a cumulative probability for chow (dashed line), HF-CM (gray line), and HF-AL (black line) groups. (c) Normalized PNN intensity expressed as a frequency distribution for chow, HF-CM, and HF-AL groups. (d) Normalized average PNN intensity among chow (white), HF-CM (gray), and HF-AL (black) groups. (e) PNN number, measured by the number of WFA+ cells. (f) Cell density, measured by the number of DAPI+ cells. (g) Representative images shown as max projections of z-stacks. Values represent the mean ± SEM (^∗^/^+^*p* < 0.05). ∗ indicates significant difference between HF-CM and chow as well as between HF-AL and chow. + indicates significant difference between HF-AL and HF-CM groups.

In the PL-PFC, the Kolmogorov-Smirnov test applied to normalized PNN intensities across experimental groups revealed a significant change in the cumulative distribution between HF-AL and chow as well as between HF-CM and chow (average PNN intensities—chow: 101.0 ± 3.40, HF-CM: 65.10 ± 1.61, and HF-AL: 58.15 ± 1.30; ^∗^*p* < 0.001, Figures 2(b) and 2(c)). A slight difference was also detected between HF-AL and HF-CM groups (^+^*p* < 0.05). One-way ANOVA revealed a significant fat-induced reduction in normalized average intensity in both HF groups compared to chow (average normalized PNN intensity—chow: 100.00 ± 8.14, HF-CM: 67.96 ± 4.08, and HF-AL: 67.20 ± 2.99; *F*_(2,20)_ = 12.16, ^∗^*p* < 0.001, Figure 2(d)). The diet has no effect on the WFA+ or DAPI+ number (Figures 2(e) and 2(f)).

**Figure 3:**
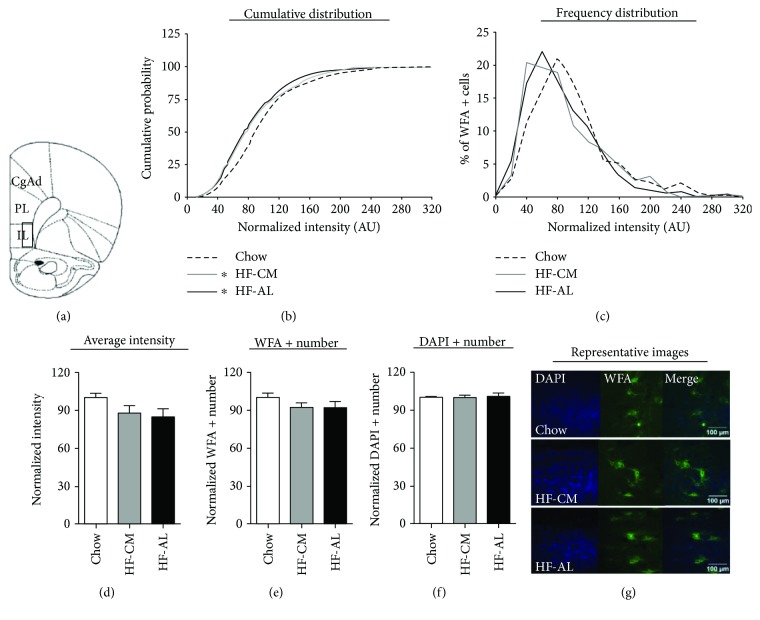
Consumption of a high-fat diet does not alter PNN intensity nor PNN number in the infralimbic prefrontal cortex (IL-PFC). (a) Schematic of the region analyzed. (b) Normalized PNN intensity expressed as a cumulative probability for chow (dashed line), HF-CM (gray line), and HF-AL (black line) groups. (c) Normalized PNN intensity expressed as a frequency distribution for chow, HF-CM, and HF-AL groups. (d) Normalized average PNN intensity among chow (white), HF-CM (gray), and HF-AL (black) groups. (e) PNN number, measured by the number of WFA+ cells. (f) Cell density, measured by the number of DAPI+ cells. (g) Representative images shown as max projections of z-stacks. Values represent the mean ± SEM (^∗^*p* < 0.05). ∗ indicates significant difference between HF-CM and chow as well as between HF-AL and chow.

In the IL-PFC, there were no observed dietary effects on PNN intensity when measured as normalized average intensity (Figure 3(d)). Similarly, there were no differences in the WFA+ or DAPI+ number (Figures 3(e) and 3(f)). However, the Kolmogorov-Smirnov test applied to normalized PNN intensities across experimental groups revealed a significant difference between HF-AL and chow as well as between HF-CM and chow (average PNN intensities—chow: 98.32 ± 2.37, HF-CM: 87.91 ± 2.14, and HF-AL: 84.64 ± 2.06; ^∗^*p* < 0.001, Figures 3(b) and 3(c)). No differences were detected between HF-AL and HF-CM.

**Figure 4:**
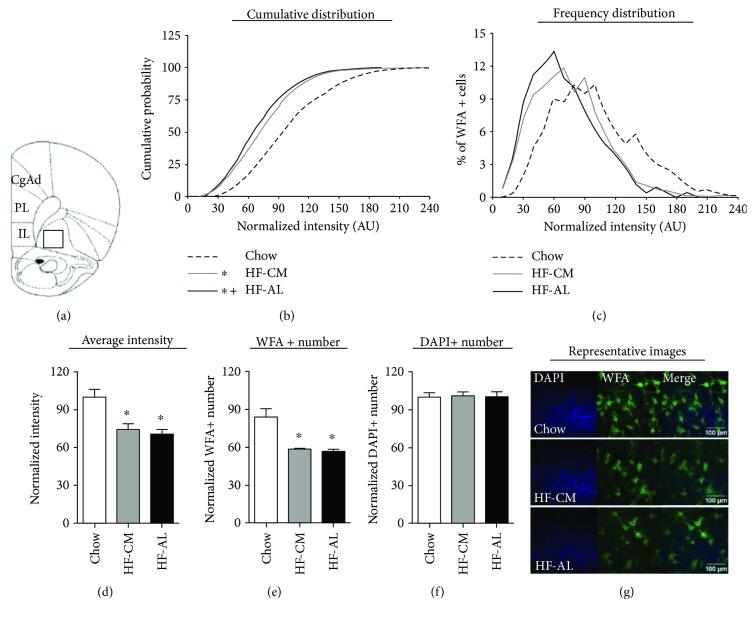
Consumption of a high-fat diet attenuates PNN intensity and number, in the orbitofrontal cortex (OFC). (a) Schematic of the region analyzed. (b) Normalized PNN intensity expressed as a cumulative probability for chow (dashed line), HF-CM (gray line), and HF-AL (black line) groups. (c) Normalized PNN intensity expressed as a frequency distribution for chow, HF-CM, and HF-AL groups. (d) Normalized average PNN intensity among chow (white), HF-CM (gray), and HF-AL (black) groups. (e) PNN number, measured by the number of WFA+ cells. (f) Cell density, measured by the number of DAPI+ cells. (g) Representative images shown as max projections of z-stacks. Values represent the mean ± SEM (^∗^/^+^*p* < 0.05). ∗ indicates significant difference between HF-CM and chow as well as between HF-AL and chow. + indicates significant difference between HF-AL and HF-CM groups.

In the OFC, the Kolmogorov-Smirnov test applied to PNN intensities across experimental groups revealed a significant difference between HF-AL and chow as well as between HF-CM and chow (average PNN intensities—chow: 99.62 ± 1.36, HF-CM: 75.07 ± 1.21, and HF-AL: 70.28 ± 1.19; ^∗^*p* < 0.001, Figures 4(b) and 4(c)). A significant difference was also detected between HF-AL and HF-CM (^+^*p* < 0.01). One-way ANOVA revealed a significant fat-induced reduction in the mean of the normalized average intensity in both HF groups compared to chow (average normalized PNN intensity—chow: 100.0 ± 6.18, HF-CM: 74.51 ± 4.50, and HF-AL: 70.13 ± 4.34; *F*_(2,20)_ = 12.16, ^∗^*p* < 0.001, Figure 4(d)). One-way ANOVA also revealed a significant fat-induced reduction in the normalized WFA+ number (average normalized WFA number—chow: 111.8 ± 9.16, HF-CM: 78.01 ± 1.16, and HF-AL: 75.05 ± 2.80; *F*_(2,20)_ = 15.07, *p* < 0.001, Figure 4(e)). The diet has no effect on the DAPI+ number (Figure 4(f)).

## 4. Discussion

Our results show for the first time that dietary fat alters PNNs in the PFC, most robustly in the PL-PFC and OFC subregions. In the PL-PFC, we observed a fat-induced reduction in PNN intensity. A recent study indicates that exposure to a high-fat diet decreases GABA concentrations in the frontal cortex [18]. Further, degradation of PNNs reduces the GABAergic cell firing rate [16] and is therefore likely to reduce regional concentrations of GABA. Thus, it is possible that the decrease in GABA concentration is in part due to the observed reduction in PNN intensity in the PL-PFC. Future studies should seek to elucidate this relationship. It is worth noting that while both fat-exposed groups were significantly different from chow with regard to PNN intensity in the PL-PFC, the Kolmogorov-Smirnov test of normalized PNN intensities detected a slight difference between HF-AL and HF-CM, suggesting that weight gain itself is perhaps mildly important in mediating these changes.

Although we did detect small differences in the cumulative distribution of normalized PNN intensities between HF-AL and chow and between HF-CM and chow in the IL-PFC, overall the PNNs of this region do not appear to be robustly affected by a HF diet. However, it is possible that the small shift in cumulative distribution may affect behaviors mediated by this region, such as extinction learning [21]. This observation was somewhat unexpected, given our previous observation that dietary fat attenuates thin spine density in this region [10]. Thus, we hypothesize that PNNs and dendritic spines do not share a simple correlative relationship.

In addition to examining the PL-PFC and IL-PFC, we also analyzed PNNs of the ventral OFC, which revealed a pronounced dietary effect on both PNN intensity and PNN number. The interpretation of these data is somewhat more complex than that in the medial PFC, in part because relative to the PL-PFC and IL-PFC, much less is known regarding the OFC in the context of obesity, inhibitory currents, and PNNs. Recent evidence demonstrates that extended access to a “cafeteria diet”, high in both fat and carbohydrates, elicits a reduction in inhibitory transmission onto *lateral* OFC pyramidal cells, due to decreased release probability of GABAergic inputs [22]. Although this study examined the effects of a slightly different high-fat diet in an adjacent region, our observations are complementary and together suggest that dietary fat promotes a reduction in GABA transmission in the OFC, which may be mediated by a loss of PNNs and/or PNN intensity. It is worth noting that acute environmental enrichment increases PNN intensity in the OFC [19]. Thus, forms of environmental enrichment, such as exercise, could be utilized to reverse fat-induced deficits in the OFC. Future studies will test this intervention strategy.

There are several caveats worth discussing. First, by nature of the experimental design, animals in this study were individually housed. This practice was used in order to measure the caloric intake from each animal as well as to ensure that animals in the HF-CM did not have to compete to receive their full allotment of food each day. Importantly, social isolation has been reported to reduce parvalbumin-containing cell density in the hippocampus [23] and reduce medial PFC volume [24]. In the present study, individual housing was applied to every animal in each experimental group. Thus, even if social isolation does affect PNNs in the PFC, it is unlikely to have affected the trends observed here. Second, the HF diet is different from the chow diet not just in the percentage of kilocalories from fat but in the percentage of kilocalories from protein and carbohydrates as well. The difference in % fat, % protein, and % carbohydrates is 47%, 9%, and 38%, respectively. Thus, it cannot be conclusively stated that the observed diet-induced changes in PNNs are a result of increased fat consumption; they could in fact be due to a reduction in protein and/or carbohydrate consumption. Finally, while it is known that the presence of PNNs affects the firing rate of the GABAergic cells they surround [16], the role that PNN intensity has in cell function has not been examined. It is therefore difficult to interpret the impact that changes in PNN intensity may have on cell firing and/or behavior until this is known. Our laboratory is currently investigating this important gap in knowledge.

The results of the present study demonstrate a novel diet-induced alteration in PNNs of the PFC in a regionally specific manner, providing further insight into fat-induced structural changes in the brain. Disruption of PNNs in the PFC, an area critical for reward-driven behavior and decision-making, may have implications for nutritional choice and other feeding behaviors. Thus, future research efforts should explore the behavioral phenotypes associated with these structural deficits in order to determine whether PNN-mediated inhibition of the PFC may serve as a potential therapeutic target in the treatment of obesity and other conditions of nutritional excess.
